# Supplementary material for: Effect of genomic and cellular environments on gene expression noise
Source: Genome Biol. 2024 May 24;25:137. doi: 10.1186/s13059-024-03277-9 (PMC11127367; doi:10.1186/s13059-024-03277-9)
Supplement: Supplementary file 1 — Additional file 1: Supplementary Figures. [file 13059_2024_3277_MOESM1_ESM.pdf]

Supplementary Figures

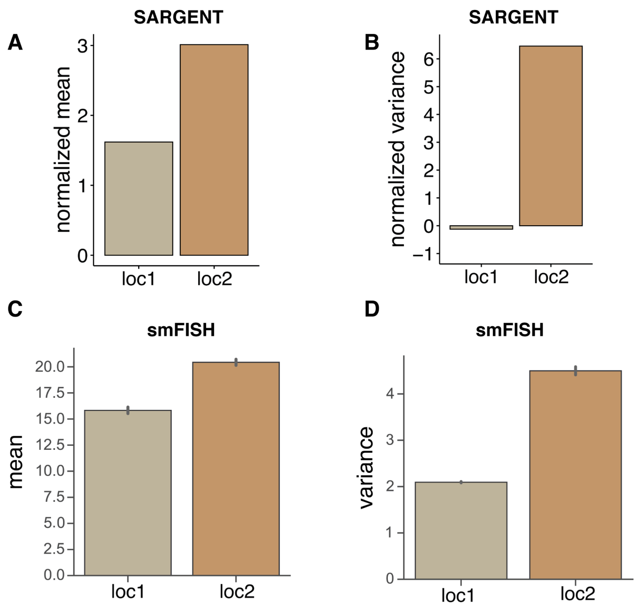

Fig S1: (A, B) Mean and noise levels of two IR locations (loc1 and loc2) measured by SARGENT. Values were normalized (z-scored) for comparison across different experiments. **(C,D)** Mean and noise levels of the same two IR locations measured with smFISH. Error bars represent 1 std from two biological replicates.

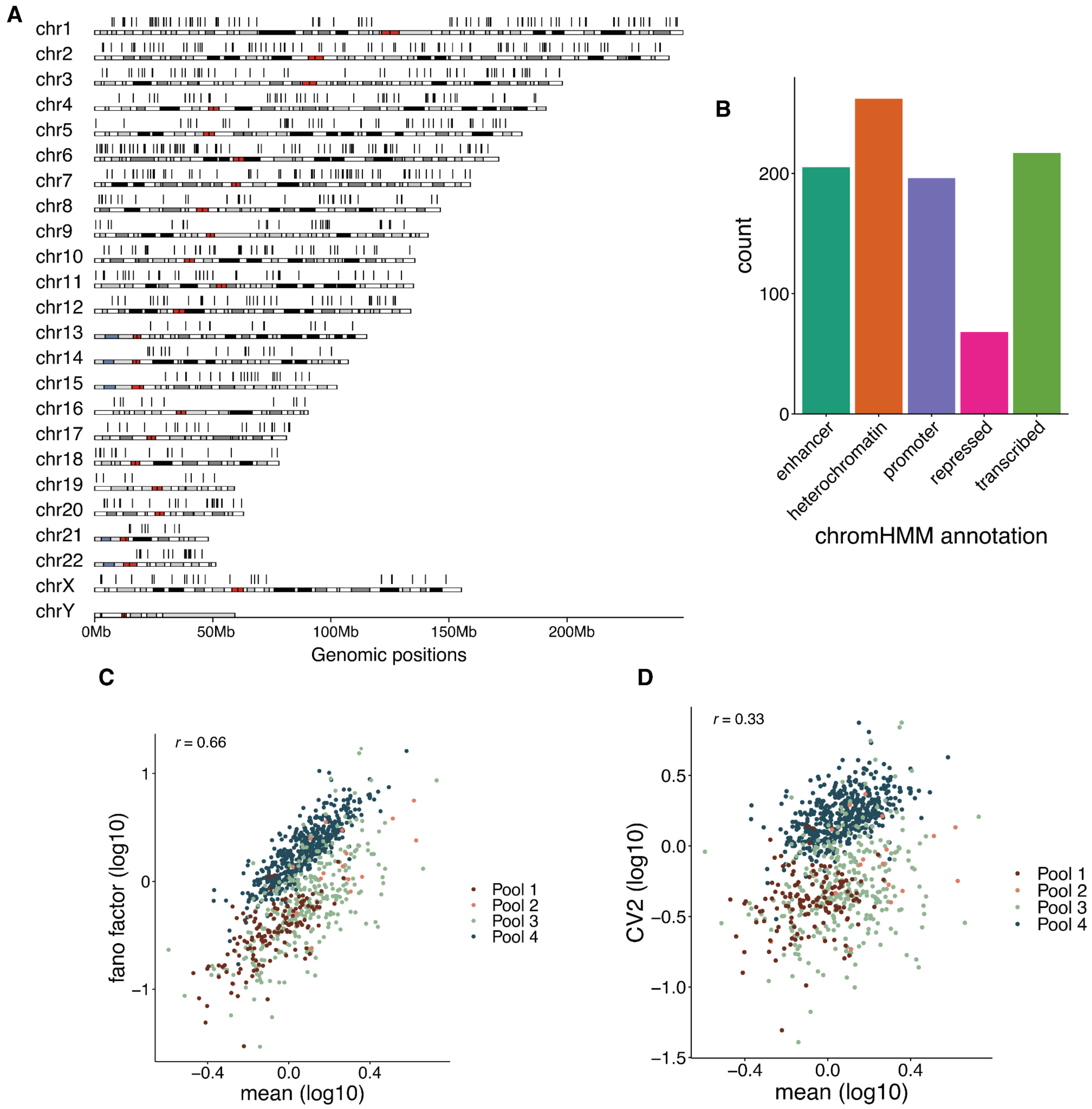

Fig S2: Measurements of mean-independent noise across different chromosomal environments. (A) IR locations are distributed all throughout the genome. Each black bar above the ideogram represents a separate integration. **(B)** IR locations are found distributed across different chromatin types. **(C, D)** Expression mean is well correlated with fano factor (C) and CV^2 (D).

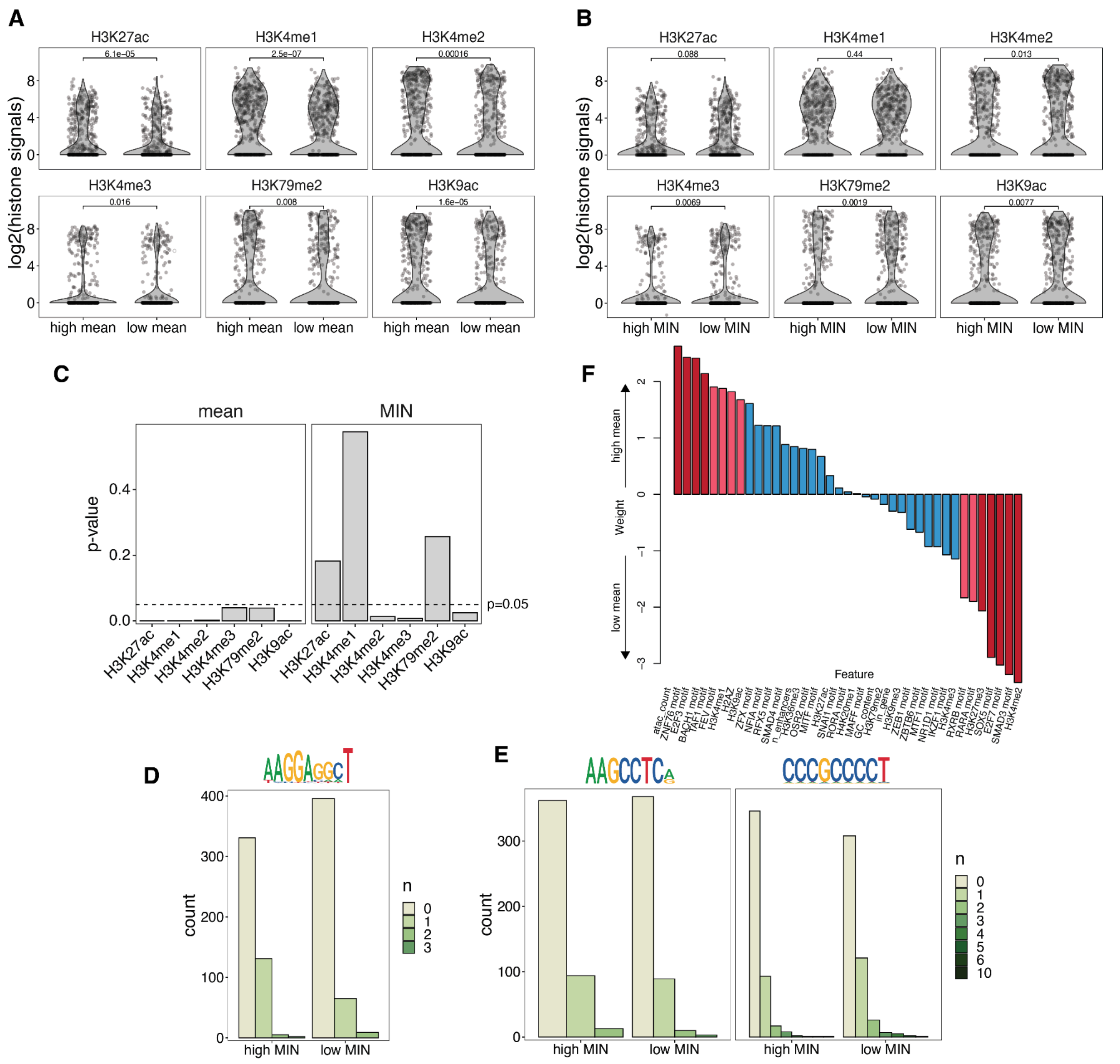

Fig S3: Different chromatin modifications distinguish high and low mean/MIN levels. (A, B) Histone signals averaged across the 10kb surrounding each IR for high vs low mean **(A)** or MIN **(B)** locations. Each point represents a single IR. p-values were calculated with a two-sided Wilcoxon test. **(C)** Empirical p-values for observed differences calculated from scrambling the mean/MIN labels 1000x. **(D, E)** Occurrence of each enriched motif for high MIN **(D)** or low MIN **(E)** from Figure 3C in the 10kb surrounding each IR. The respective motifs are indicated above each panel. **(F)** Weights of features from the gene expression mean model using only intrinsic genomic features. Red bars: p-value < 0.05; Pink bars: 0.05 < p-value < 0.1 from the logistic regression model.

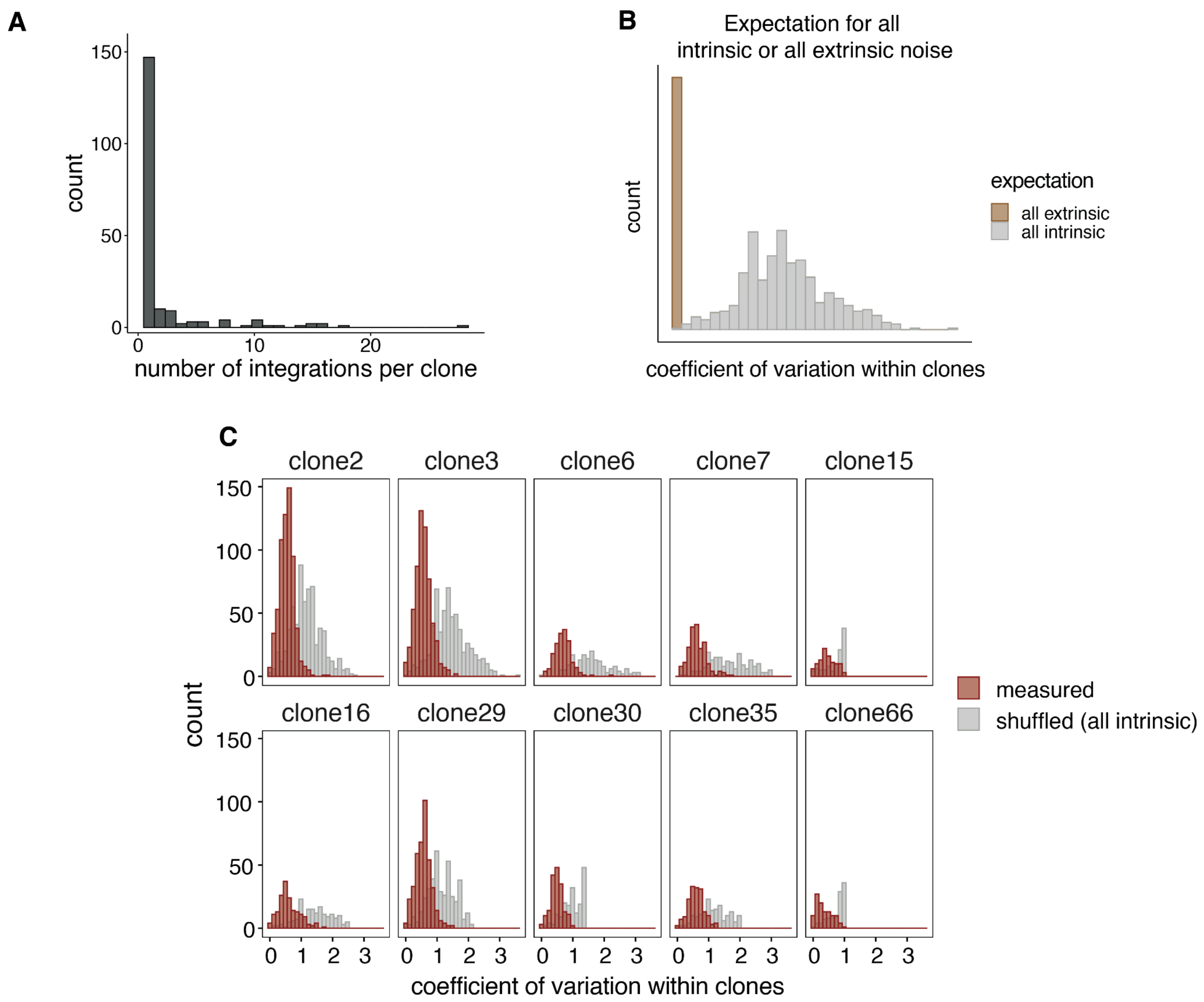

Fig S4: Clonal identification allows for separation of intrinsic and extrinsic noise. (A) Histogram of integrations per clone. **(B)** Mock histogram showing the expected distributions if noise was either all intrinsic or all extrinsic. **(c)** Histogram of measured coefficient of variation for 10 random clones. The shuffled distribution represents the distribution after cell labels have been shuffled, which simulates the case when all noise is intrinsic.

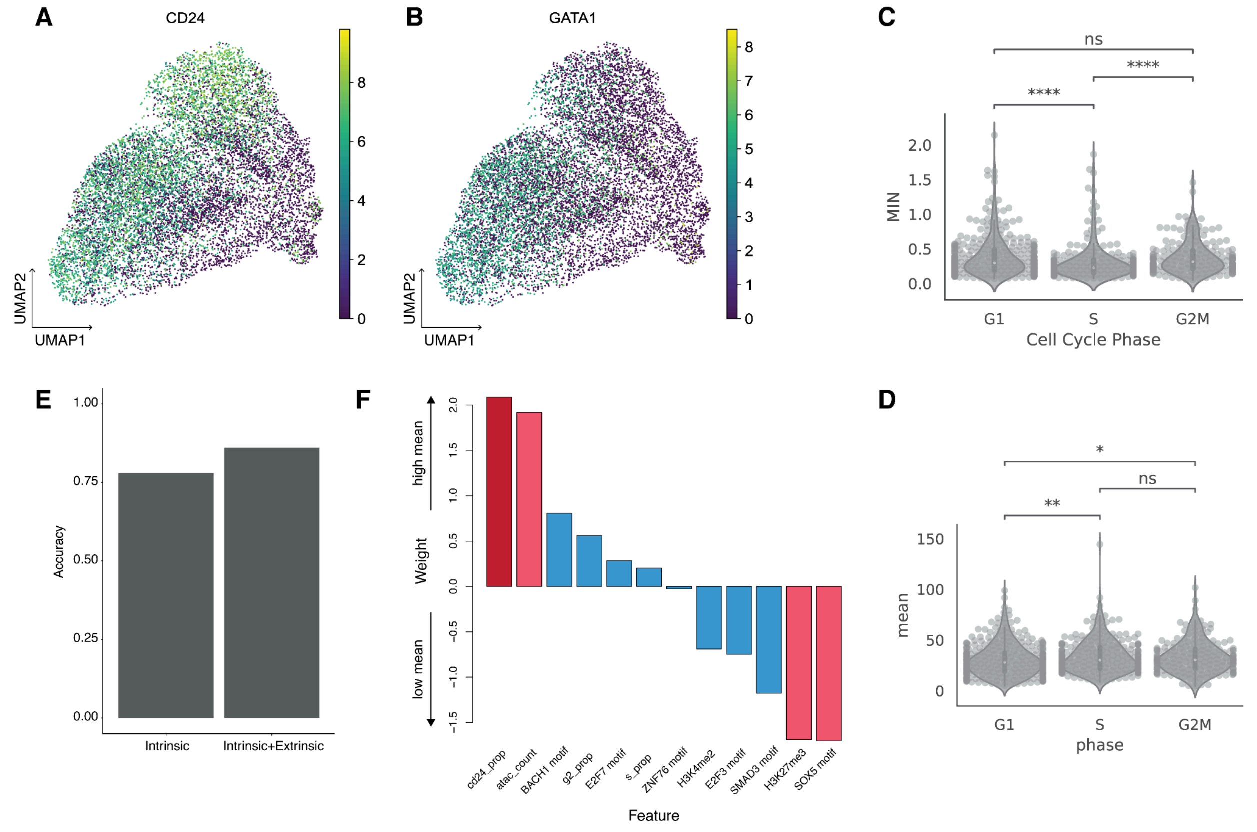

Fig S5: Cell cycle and CD24 states partially explain extrinsic noise. (A) UMAP clustering reveals CD24⁺ cell population. **(B)** CD24⁺ cells express stemness and proliferation marker gene GATA1. **(C, D)** Violin plots of MIN (C) and mean (D) levels in different phases of the cell cycle. P-values were calculated using the Mann-Whitney-Wilcoxon test. Legend: *: 0.01 < p-value ≤ 0.05, **: 0.001 < p-value ≤ 0.01, ***: 0.0001 < p-value ≤ 0.001, ****: p-value ≤ 0.0001. **(E)** Gene expression mean model improves after the addition of extrinsic features. **(F)** Weights of features from the mean model using both intrinsic genomic and extrinsic features. Red bars: p-value < 0.05; Pink bars: 0.05 < p-value < 0.1 from the logistic regression model.

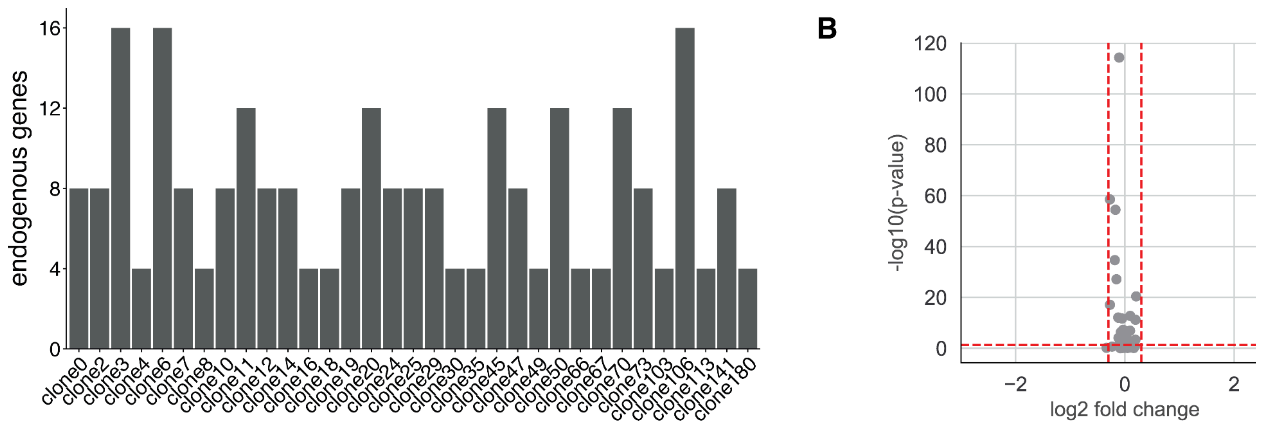

Fig S6: IR integrations have little impact on endogenous expression. (A) Bar plot number of IRs in endogenous genes per clone. **(B)** Shuffling IR-endogenous gene labels results in no differentially expressed genes.
