# Supplementary material for: Effect of genomic and cellular environments on gene expression noise
Source: Genome Biol. 2024 May 24;25:137. doi: 10.1186/s13059-024-03277-9 (PMC11127367; doi:10.1186/s13059-024-03277-9)
Supplement: Supplementary file 11 — Additional file 11: Review history. [file 13059_2024_3277_MOESM11_ESM.docx]

Review History

**First round of review**

**Reviewer 1**

**Were you able to assess all statistics in the manuscript, including the appropriateness of statistical tests used?**

Yes, and I have assessed the statistics in my report.

**Comments to author:**

Hong et al. presented a high throughput tool for measuring mean expression and expression noise using random integration of a cell reporter system. Successful random integration of the reporter, mediated by piggyBac transposes, leads to the expression of a fluorescent protein under the control of a CMV promoter. The reporter sequence contains location barcodes (loc BC), locating genomic integration sites, and capture sequences (CS), quantifying RNA expression, which enables measuring individual cells' expression level and noise. The authors conclude that the chromatin state at the integration site contributes to intrinsic noise and the cell state to extrinsic noise. Overall expression noise results from the nearly equal contribution of intrinsic and extrinsic noises.

The approach proposed in the manuscript is sensible and novel. The manuscript is well written, with few exceptions, having a smooth flow making it easy to understand. Using "MIN" metric for measuring the expression noise by regressing out the driving effect of mean on expression seems rational and practical. Authors use multiple models, statistical frameworks, and analysis to predict the effect of different factors on low and high MIN. My main confusions are around this part, needing more description of models, including metrics, parameters, robustness, and discussion on the model predictions or comparison between different outcomes in detail. I have listed my specific concerns/comments below.

Concerns:

1. Generation of cell lines for SARGENT. The manuscript says "The SARGENT library and piggyBac transposase were co-transfected into K562 (LP cell lines) cells at a 3:1 ratio using the Neon Transfection System". I couldn't find number of transfections used for making pools. What was the transfection efficiency? Having a figure showing the average number of successful clones (GFP+) per transfection before cell sorting and the average number of unique integrations in each clone after sorting can help future readers determine whether they want to attempt this experiment. This is particularly important for increasing the chance of reproducibility of results and experimental design for potential users.

2. Incomplete description of methods/motivations for models/omics data. TF binding and chromatin are the features investigated for possible sources of intrinsic noise, but the motivations for doing so are unclear. Were all features included as potential features when training intrinsic noise models? Was the availability of data on these two factors the compelling reason to use them? Could other factors like presence of enhancers and promoters around integration sites change the model prediction or score? In the method section mentions about K562-Hi-C on wildtype cells but I couldn't find any results or analysis exploring the data in the text.

3. Issue with Fig 3A,B. What is the y axis unit for A and B? I think the point of fig 3 A B is to suggest that there may be chromatin marks that can distinguish high and low mean/MIN. However, how much of the signal we are seeing here could result from outliers that skew the distribution of either high or low sets? If we are looking at something like a mean, this is possible.

4. Fig 4C analysis. The 192 clones selected for analysis are from pool 4? the number of integrations per clone with mean of 3 per clones was the highest integration number achieved in this study? Each dot in the figure represent one pairwise expression for one cell? What causes the vertical and horizontal lines in this graph (sets of dots that form a row or column? How many cells in total? Does each cell contribute similarly to this plot? More details on the statistical framework used here would be helpful.

5. Fig 5 is somewhat confusing. "The stem-like substate is marked by high CD24 expression and proliferates at a higher rate, which we hypothesized would contribute to extrinsic noise. This hypothesis predicts that the same IRs will have higher MIN in stem-like cells compared to more differentiated cells". This contradicts the later statement in the text, "We then calculated the expression mean and MIN for each IR location separately in the two substates. IR locations in the stem-like substate have higher mean and lower MIN (Figures 5A, 5B)". It is unclear what is shown in Fig5B. To my eye, the MIN looks nearly identical for CD24+ and diff states. If I use a ruler to compare, the median is lower for CD24, contradicting the original hypothesis (but consistent with the second quoted line). Given the difficulty in seeing any difference in Fig 5B, maybe presenting this in a different way would be better?

**Reviewer 2**

**Were you able to assess all statistics in the manuscript, including the appropriateness of statistical tests used?**

Yes, and I have assessed the statistics in my report.

**Comments to author:**

Transparency statement: this review was written by Guillaume Filion (University of Toronto). I accepted the assignment on December 23, 2022. On January 5 I contacted the Editor to request access to the GEO records of the study. The link to the GEO records was made available to me on February 6 and I turned in my review on February 14. I worked approximately 12 hours on the review. I feel qualified to review the manuscript and I declare no conflict of interest.

In the manuscript titled "Effect of genomic and cellular environments on gene expression noise", Hong, Ramu, Zhao and Cohen present a technology to set up single-cell integrated reporters allowing them to probe the effect of the genomic context (the integration site in this case) on the cell-to-cell variability in gene expression. In a nutshell, the method is to insert reporters expressing a barcoded transcript that is different for every insertion, and to perform single-cell sequencing with the 10X technology. This research line is conceptually important because position effects — the fact that gene expression depends on the position in the genome — were initially discovered in the 1930s from increased expression noise. However, this specific aspect is still not explained so any progress in this regard is welcome.

I think the design of the SARGENT assay is good because it is relatively simple, considering the difficulty of the task. Overall I think that the article is interesting and a good match for the readership of Genome Research.

That said, I have doubts about the validity of the analyses. Before explaining what they are, I would like to emphasize that this is not an attack. The questions below are genuine and I am interested in the point of view of the Authors. If they can justify their approach: great. Otherwise, I hope that they will take this discussion as a guide to improve the quality of the manuscript.

MAJOR ISSUES (from most to least severe)

1. What does MIN measure exactly? The residuals of a regression line are often interpreted as error. Figures 2A and 2B show that the uncertainty around the log(mean) is around 0.3 while that around log(variance) is around 1. So the position of the points away from the regression line in Figure 2C have a random component due to measurement errors. Does MIN actually measure experimental noise? Given its importance for the rest of the manuscript, the Authors should calibrate their score against some standard and see that it meets some expectation. This is mission-critical: when coming up with new measurement methods, one has to test the validity of the measurements.

2. Figure 2C shows that log(variance) depends linearly on log(mean) with a slope approximately equal to 2. However, the intercept varies between replicates: for any average expression, the variance in Pool 4 is about 3 times higher than in Pool 1. Why is it the case? To me the measurements do not seem so reproducible between experiments.

A common observation is that the expression of integrated transgenes varies over time in the first few weeks. Were the measurements made at the same time after insertion? If not, the pools may be at different stages of epigenetic silencing (for example) with different noise features.

3. Page 6, line 54. "... achieved an accuracy of 82% on the training data. When applied to holdout data, the trained model achieved 62% accuracy". See that the accuracy drops by 20% between training and testing, which suggests overfitting. Also observe that 62% means practically no predictive power, given that the minimum is 50% for binary predictions, even more if labels are unbalanced.

Page 10, line 21 "the model achieved 75% accuracy (albeit without a holdout set to test on due to the small number of locations with associated extrinsic features)". The reported accuracy might be no better than random. Having no test set is not an option here because of the risk of overfitting reported above. The authors could perform leave-one-out cross validation to estimate the predictive power of their model.

4. It is difficult to understand how robust the motifs of Figure 3C are. If the labels high MIN and low MIN are assigned randomly, would the Authors discover similar motifs? Also, what is the occurrence of the motifs in the search regions? It would be useful to show the percentage of regions that have the motif (counting the regions just once if they overlap between different insertions).

5. Why do the plots of Figures 3A and 3B show peaks in every panel? In other words, why would it be the case that the average signal of every histone modification is lower 5 kb upstream and downstream of every insertion point? Is this because only marks associated with activation are shown? Also, why not show marsk associated with repression?

6. Page 18, line 37. "For each locBC, mean expression was calculated as the average UMI count across all cells that express that locBC." The minimum mean expression is 1, with a log equal to 0. However, in Figures 2A, 2C and 2D there appears negative logs, meaning that the mean is less than 1. How are the means computed exactly? Was some centering applied, and if so, why would it not be the same mean between replicates?

MISSING INFORMATION (from most to least severe)

1. I could not find the code to reproduce the analyses of the manuscript. Details matter for this work, so it is important to release the code that was used (not just the software tool).

2 . What are the definitions of high and low MIN? High and low mean?

3. Page 15, line 21: "3µg of transposase". Is this really the transposase, or do the authors mean a transposase-expressing vector or a transposase mRNA? If this is the transposase, what is the origin or how was it purified? If this is a transposase-expressing vector, which is it in particular? If a transposase mRNA, how was it prepared and from which vector?

4. Page 17, line 47: "fuzzy match algorithm". Which one? Do the authors just mean that they allowed a single mismatch (but no insertion or deletion) in the constant region of the read? This could be clarified.

5. Page 17, line 48: "we parsed out the cell barcode". By absolute position? By position relative to the constant region? By another method? Indels are very rare in Illumina reads, but the locBCs were random oligos, where indels are not so rare. The details could matter here.

MINOR ISSUES

1. Page 3, line 10: "We chose to use the CMV promoter because it should be independent of the cell's regulatory networks and therefore should not be affected by endogenous cellular feedback loops". A citation is in order. Also, is this really reasonable to imagine that the CMV is not impacted by the cell state?

2. Page 8, line 29. "We calculated the standard deviation relative to the mean of all IRs in that cell, which we define as the fluctuation index." This is widely known as the coefficient of variation. Why not call it the coefficient of variation?

3. Page 17, line 51: "we filtered out trios with low numbers of supporting reads". What is low? Also, what is the evidence that such trios are likely to be enriched for PCR artefacts? If the average number of reads per mRNA is substantially higher, then indeed, a number of reads much below average is suspicious and probably due to PCR or sequencing artifacts. But if the average number of reads per mRNA is low, then the argument for discarding those trios could become invalid. Therefore, the authors should indicate what the average number of reads per mRNA is and use it to justify the threshold that was chosen for "low".

Page 18, line 12: "We recovered ~12% the reads that would have been discarded". Why throw away the trios with few reads then? If the error correction scheme is good, wouldn't it make sense to rescue the trios with few reads?

4. Page 18, line 4: "We found that 99% of 10x cell barcode are more than 6 hamming distances away from each other". Does this mean that 99% of the pairs have a Hamming distance above 6, or that 99% of the barcodes have a Hamming distance above 6 against every other barcode? Or does it mean something else?

5. Page 18, line 17: "all the locBCs are ranked based on abundance". Is this the abundance of the number of reads, the number of UMIs? Another abundance?

6. Page 18, line 29: "We filtered out cells that had less than 5 IR intergrations (locBCs) and less than 10 UMIs". Does this mean that a cell with fewer than 5 integrated reporters is systematically thrown away for the rest of the study? This seems implausible since the authors report a mean of 3 integrations per clone at page 8, line 17. Maybe the authors mean that some filtering of the locBCs was performed in the transcriptome? In this case, it is not clear what this means, as counting could be based on the reads, the UMIs, or something else.

7. Page 18, line 33: "We also filtered out locBCs that were seen in less than 5 cells". The justification that these filters were chosen to maximize reproducibility between replicates would require some data showing that those numbers reach an optimum of reproducibility (to be further defined). Meanwhile, removing locBCs with low expression introduces a bias in the mean. This should be discussed and justified.

8. Page 18, line 37: "mean expression was calculated as the average UMI count across all cells that expressed that locBC". The estimator is biased because it does not take into account cells where the locBC is present but not expressed. The authors know which barcodes are present but not expressed in a given cell, as they explain page 8, line 16. The unbiased estimator would be based on a larger sample size, so it seems a better choice to me. The same applies to the estimator of the variance.

COMMENTS — nothing to address, just for the sake of scientific discussion.

1. Page 18, line 16-21: Work by Björklund (https://pubmed.ncbi.nlm.nih.gov/27874090/) showed that this variant of sequence clustering seems to be less effective than another one referred to as "message passing". The authors may be interested in some earlier discussion on the design of barcodes and error-correction algorithms.

2. Page 15, line 57: "For each pool, we performed 2 technical replicates with 8 PCR per replicate". How much input DNA is in each PCR? The weight of one haploid human genome is approximately 35 pg, so for a population of dividing diploid cells, the DNA of one cell is approximately 100 pg. A PCR with 3 ng of input thus corresponds to ~30 cells, carrying at most 150 insertions (based on the assumption of the authors that each cell contains 5 insertions). In practice, inverse-PCR loses at least 2/3 of the insertions because the T4 DNA ligase reaction is far from complete in 16 hours, and some insertions are lost because the restriction fragment is either too large or too small. Thus, a set of 8 PCRs with 3 ng each carries at most ~500 target DNA molecules that can be amplified. For the declared target of 500 insertions per pool, this is a 1x coverage.

It is important to know whether the input in each PCR is above or below 3 ng to know if the coverage is sufficient to discover most of the insertions, or if the mapping explores only a small fraction of the insertions.

**Authors Response**

**Point-by-point responses to the reviewers’ comments:**

Reviewer #1:

Hong et al. presented a high throughput tool for measuring mean expression and expression noise using random integration of a cell reporter system. Successful random integration of the reporter, mediated by piggyBac transposes, leads to the expression of a fluorescent protein under the control of a CMV promoter. The reporter sequence contains location barcodes (loc BC), locating genomic integration sites, and capture sequences (CS), quantifying RNA expression, which enables measuring individual cells' expression level and noise. The authors conclude that the chromatin state at the integration site contributes to intrinsic noise and the cell state to extrinsic noise. Overall expression noise results from the nearly equal contribution of intrinsic and extrinsic noises.

The approach proposed in the manuscript is sensible and novel. The manuscript is well written, with few exceptions, having a smooth flow making it easy to understand. Using "MIN" metric for measuring the expression noise by regressing out the driving effect of mean on expression seems rational and practical. Authors use multiple models, statistical frameworks, and analysis to predict the effect of different factors on low and high MIN. My main confusions are around this part, needing more description of models, including metrics, parameters, robustness, and discussion on the model predictions or comparison between different outcomes in detail. I have listed my specific concerns/comments below.

*Reply: We thank the reviewer for the positive assessment of our work.*

Concerns:

1. Generation of cell lines for SARGENT. The manuscript says "The SARGENT library and piggyBac transposase were co-transfected into K562 (LP cell lines) cells at a 3:1 ratio using the Neon Transfection System". I couldn't find number of transfections used for making pools. What was the transfection efficiency? Having a figure showing the average number of successful clones (GFP+) per transfection before cell sorting and the average number of unique integrations in each clone after sorting can help future readers determine whether they want to attempt this experiment. This is particularly important for increasing the chance of reproducibility of results and experimental design for potential users.

*Reply: The text now states that we performed two transfections each time we made a pool of cells. We generally obtain >50% positive cells when sorting. When measured by qPCR, we obtain on average 10 integrations per clone, though this number varies by clone from about 1-20+ as seen in Supplementary Fig 4A. As these numbers will vary based on the chosen cell lines and transfection methods, we did not include the flow cytometry plot. This part of the pool generation is also based on the TRIP method published in Akhtar et al. (PMID: 23953119), suggesting that this is a robust protocol across labs.*

2. Incomplete description of methods/motivations for models/omics data. TF binding and chromatin are the features investigated for possible sources of intrinsic noise, but the motivations for doing so are unclear. Were all features included as potential features when training intrinsic noise models? Was the availability of data on these two factors the compelling reason to use them? Could other factors like presence of enhancers and promoters around integration sites change the model prediction or score? In the method section mentions about K562-Hi-C on wildtype cells but I couldn't find any results or analysis exploring the data in the text.

*Reply: Besides chromatin and TF binding, we also considered the presence of enhancers that looped to each location (derived from the Hi-C data), GC content, number of ATAC-seq peaks around the IRs and whether or not the IR was in a gene as potential sources of intrinsic noise in the model.*

*We have clarified this point in the main text (lines 198-201) and Methods section as well (lines 653-666). The only feature that contributed significantly to the model is whether or not the IR is in a gene. We also note that features such as the number of enhancers/promoters around the gene is generally correlated with the levels of H3K27ac and H3K4me3 respectively, and would therefore introduce more correlating variables into the model.*

3. Issue with Fig 3A,B. What is the y axis unit for A and B? I think the point of fig 3 A B is to suggest that there may be chromatin marks that can distinguish high and low mean/MIN. However, how much of the signal we are seeing here could result from outliers that skew the distribution of either high or low sets? If we are looking at something like a mean, this is possible.

*Reply: The y axis units are averaged from ChIP-seq signals using ComplexHeatmap. To check for outliers, we also calculated the cumulative (average) histone signals per region and plotted them as violin plots. We do not observe any outliers that seem to skew the distribution in all sets of locations (see figure below). We also randomly permuted the labels for high/low mean and MIN to calculate p-values for the difference we observed, and these p-values agree with the observed differences in Figure 3A and 3B. We have included this analysis in Supplementary Figures 3A-3C (lines 172-175).*

4. Fig 4C analysis. The 192 clones selected for analysis are from pool 4? the number of integrations per clone with mean of 3 per clones was the highest integration number achieved in this study? Each dot in the figure represent one pairwise expression for one cell? What causes the vertical and horizontal lines in this graph (sets of dots that form a row or column? How many cells in total? Does each cell contribute similarly to this plot? More details on the statistical framework used here would be helpful.

*Reply: We have now clarified the text to address this point. The 192 clones selected for analyses are indeed from pool 4. The number of integrations identified per clone ranges from 1-27, which we show in Supplementary Figure 4A. The mean appears low because we were unable to recover every barcode from each clone or could not assign barcodes to any clone due to dropout from 10x sequencing. Each dot in Figure 4C represents a pair of barcodes (IRs) from the same cell, but multiple pairs of barcodes from the same cell can be plotted here. If the pairwise expression of IRs is generally well correlated, that indicates that expression of the two IRs is co-fluctuating and suggests that extrinsic noise is the major driver of expression noise. However, if the two IRs are very different, the expression of the two IRs are independent, suggesting that intrinsic noise at each IR location is the major driver of intrinsic noise. We have clarified this in the figure legend (line 275-277) and changed the axes of Figure 4C to be more informative. These two axes can be separated using the statistical framework developed by Fu and Pachter (PMID: 27875323)****.*** *We have provided details on the framework in the Methods (lines 722-728).*

5. Fig 5 is somewhat confusing. "The stem-like substate is marked by high CD24 expression and proliferates at a higher rate, which we hypothesized would contribute to extrinsic noise. This hypothesis predicts that the same IRs will have higher MIN in stem-like cells compared to more differentiated cells". This contradicts the later statement in the text, "We then calculated the expression mean and MIN for each IR location separately in the two substates. IR locations in the stem-like substate have higher mean and lower MIN (Figures 5A, 5B)". It is unclear what is shown in Fig5B. To my eye, the MIN looks nearly identical for CD24+ and diff states. If I use a ruler to compare, the median is lower for CD24, contradicting the original hypothesis (but consistent with the second quoted line). Given the difficulty in seeing any difference in Fig 5B, maybe presenting this in a different way would be better?

*Reply: We thank the reviewer for catching this mistake, indeed, MIN is generally lower in CD24+ cells. Thus, our original hypothesis was wrong, and we have clarified this in the manuscript (lines 291292).*

Reviewer #2: Transparency statement: this review was written by Guillaume Filion (University of

Toronto). I accepted the assignment on December 23, 2022. On January 5 I contacted the Editor to request access to the GEO records of the study. The link to the GEO records was made available to me on February 6 and I turned in my review on February 14. I worked approximately 12 hours on the review. I feel qualified to review the manuscript and I declare no conflict of interest.

In the manuscript titled "Effect of genomic and cellular environments on gene expression noise", Hong, Ramu, Zhao and Cohen present a technology to set up single-cell integrated reporters allowing them to probe the effect of the genomic context (the integration site in this case) on the cell-to-cell variability in gene expression. In a nutshell, the method is to insert reporters expressing a barcoded transcript that is different for every insertion, and to perform single-cell sequencing with the 10X technology. This research line is conceptually important because position effects — the fact that gene expression depends on the position in the genome — were initially discovered in the 1930s from increased expression noise. However, this specific aspect is still not explained so any progress in this regard is welcome.

I think the design of the SARGENT assay is good because it is relatively simple, considering the difficulty of the task. Overall I think that the article is interesting and a good match for the readership of Genome Biology.

That said, I have doubts about the validity of the analyses. Before explaining what they are, I would like to emphasize that this is not an attack. The questions below are genuine and I am interested in the point of view of the Authors. If they can justify their approach: great. Otherwise, I hope that they will take this discussion as a guide to improve the quality of the manuscript.

*Reply: We thank the reviewer for the overall positive assessment of our work. We hope that the clarifications and changes provided below sufficiently address the reviewer’s concerns.*

MAJOR ISSUES (from most to least severe)

1. What does MIN measure exactly? The residuals of a regression line are often interpreted as error. Figures 2A and 2B show that the uncertainty around the log(mean) is around 0.3 while that around log(variance) is around 1. So the position of the points away from the regression line in Figure 2C have a random component due to measurement errors. Does MIN actually measure experimental noise? Given its importance for the rest of the manuscript, the Authors should calibrate their score against some standard and see that it meets some expectation. This is mission-critical: when coming up with new measurement methods, one has to test the validity of the measurements.

*Reply: Expression noise is generally computed as the Fano factor or CV^2^ - where expression noise is computed as variance/mean or variance/mean^2. As we show in Supplementary Figures 2C and 2D, both these metrics are insufficient to correct for the effect of the mean. To capture the variability not explained by the mean, we regressed out the effect of the mean. This residual variability will encompass both biological variability independent of the mean and technical noise. To ensure that the MIN metric is valid, we computed the technical reproducibility of MIN using the technical replicates described in Figure 1. This is shown in the figure below where the Pearson correlation coefficient between replicates is 0.21. Therefore, we performed our analyses not on absolute MIN levels but by binning locations into high and low MIN (top and bottom 20% of locations). To validate this approach we tested the concordance of the binning procedure across the two replicates, i.e is the same location high/low MIN in both replicates. We found that 73% of the locations that were identified as high/low MIN in one replicate were concordant in the other replicate, indicating that MIN is a reproducible metric of biological noise. In addition, we also performed smFISH to confirm the MIN trend across two locations as shown in Supplemental Figure 1.*

2. Figure 2C shows that log(variance) depends linearly on log(mean) with a slope approximately equal to 2. However, the intercept varies between replicates: for any average expression, the variance in Pool 4 is about 3 times higher than in Pool 1. Why is it the case? To me the measurements do not seem so reproducible between experiments.

*Reply: We believe that the differences between the pools is largely due to batch effects in single-cell experiments. These effects can usually be corrected using whole-transcriptome data, however, because we only sequenced reporter genes, it is difficult to accurately correct. Additionally, once we corrected to get MIN levels, these batch effects are no longer present as shown in Figure 2D, thus, we do not believe that the differences significantly change the interpretations of our results.*

A common observation is that the expression of integrated transgenes varies over time in the first few weeks. Were the measurements made at the same time after insertion? If not, the pools may be at different stages of epigenetic silencing (for example) with different noise features.

*Reply: The measurements were not all made at the same time after transgene integration. It is possible that the transgenes are silenced over time, however, piggyBac is biased for active chromatin regions and few of our locations contain repressive histone modifications (see point 5 below), reducing the likelihood of transgene silencing.*

3. Page 6, line 54. "... achieved an accuracy of 82% on the training data. When applied to holdout data, the trained model achieved 62% accuracy". See that the accuracy drops by 20% between training and testing, which suggests overfitting. Also observe that 62% means practically no predictive power, given that the minimum is 50% for binary predictions, even more if labels are unbalanced.

Page 10, line 21 "the model achieved 75% accuracy (albeit without a holdout set to test on due to the small number of locations with associated extrinsic features)". The reported accuracy might be no better than random. Having no test set is not an option here because of the risk of overfitting reported above. The authors could perform leave-one-out cross validation to estimate the predictive power of their model.

*Reply: We thank the reviewer for the helpful suggestion. We have now used leave-one-out crossvalidation (LOOCV) to estimate the model performance and replaced the previous results with the LOOCV results. These are described in Lines 201, 216, 313 and 327.*

4. It is difficult to understand how robust the motifs of Figure 3C are. If the labels high MIN and low MIN are assigned randomly, would the Authors discover similar motifs? Also, what is the occurrence of the motifs in the search regions? It would be useful to show the percentage of regions that have the motif (counting the regions just once if they overlap between different insertions).

*Reply: We randomly subsampled the motifs into 2 groups and performed the same enrichment analysis, but did not find the same motifs enriched (see figure below). For the occurrence of motifs in the search regions, we plotted the occurrences of each motif for high/low MIN sequences. Depending on the motif, each motif can occur ~0-5 times in each region. Motifs enriched in high MIN tend to have more high MIN sequences containing the motif and slightly more sequences per motif, and vice versa for low MIN motifs. We have included this analysis in Supplementary Fig 3D, 3E and lines 189-196.*

5. Why do the plots of Figures 3A and 3B show peaks in every panel? In other words, why would it be the case that the average signal of every histone modification is lower 5 kb upstream and downstream of every insertion point? Is this because only marks associated with activation are shown? Also, why not show marks associated with repression?

*Reply: The piggyBac transposase has a bias towards active chromatin regions (PMID 28252665), resulting in peaks for all the active histone modifications. Because we were looking for modifications that control transcriptional noise, we chose to use piggyBac transposase to specifically target active chromatin regions rather than repressed regions where transcription generally does not occur. As a result, we have very few integrations with repressive marks such as H3K27me3 and H3K9me3 (see figure below), giving us very little power to draw conclusions about these modifications. We have added an explanation for our choice of piggyBac in the manuscript (lines 96-98).*

6. Page 18, line 37. "For each locBC, mean expression was calculated as the average UMI count across all cells that express that locBC." The minimum mean expression is 1, with a log equal to 0. However, in Figures 2A, 2C and 2D there appears negative logs, meaning that the mean is less than 1. How are the means computed exactly? Was some centering applied, and if so, why would it not be the same mean between replicates?

*Reply: We normalize the UMI counts to correct for cell-to-cell variability in capture efficiency, this results in negative logs for some locations. We have clarified this on Lines 607-611.*

MISSING INFORMATION (from most to least severe)

1. I could not find the code to reproduce the analyses of the manuscript. Details matter for this work, so it is important to release the code that was used (not just the software tool).

*Reply: We have included the analysis code used in this study as a separate folder on our Github repository that is accessible here-* [*https://github.com/barakcohenlab/castools/tree/main/analysiscode*](https://github.com/barakcohenlab/castools/tree/main/analysis-code)

2. What are the definitions of high and low MIN? High and low mean?

*Reply: We have defined high and low MIN, high and low mean in Lines 628, 673 and 680. For the chromatin analyses, we defined high/low MIN as top and bottom 50%. To gain more power for the model we defined high/low MIN as top and bottom 20% of locations since we are more certain of the classification for these locations.*

3. Page 15, line 21: "3µg of transposase". Is this really the transposase, or do the authors mean a transposase-expressing vector or a transposase mRNA? If this is the transposase, what is the origin or how was it purified? If this is a transposase-expressing vector, which is it in particular? If a transposase mRNA, how was it prepared and from which vector?

*Reply: We apologize for the confusion - we used 3ug of a vector that expresses piggyBac transposase.*

*The transposase vector (mPBase-L3-ERT2) was obtained from Robi Mitra lab (PMID 28082389), who obtained it from Dr Allen Bradley (PMID 17576687). This has been clarified in lines 455-458. The plasmid was propagated with standard molecular biology techniques and maxiprepped for transfection.*

4. Page 17, line 47: "fuzzy match algorithm". Which one? Do the authors just mean that they allowed a single mismatch (but no insertion or deletion) in the constant region of the read? This could be clarified.

*Reply: We have clarified the details of the fuzzy match algorithm on Lines 563-564.*

5. Page 17, line 48: "we parsed out the cell barcode". By absolute position? By position relative to the constant region? By another method? Indels are very rare in Illumina reads, but the locBCs were random oligos, where indels are not so rare. The details could matter here.

*Reply: We have clarified the way we parsed out the sequence on line 566.*

MINOR ISSUES

1. Page 3, line 10: "We chose to use the CMV promoter because it should be independent of the cell's regulatory networks and therefore should not be affected by endogenous cellular feedback loops". A citation is in order. Also, is this really reasonable to imagine that the CMV is not impacted by the cell state?

2. Page 8, line 29. "We calculated the standard deviation relative to the mean of all IRs in that cell, which we define as the fluctuation index." This is widely known as the coefficient of variation. Why not call it the coefficient of variation?

*Reply: We have changed “fluctuation index” to CV in line 253 and in Supplementary Figure 4.*

3. Page 17, line 51: "we filtered out trios with low numbers of supporting reads". What is low? Also, what is the evidence that such trios are likely to be enriched for PCR artefacts? If the average number of reads per mRNA is substantially higher, then indeed, a number of reads much below average is suspicious and probably due to PCR or sequencing artifacts. But if the average number of reads per mRNA is low, then the argument for discarding those trios could become invalid. Therefore, the authors should indicate what the average number of reads per mRNA is and use it to justify the threshold that was chosen for "low".

Page 18, line 12: "We recovered ~12% the reads that would have been discarded". Why throw away the trios with few reads then? If the error correction scheme is good, wouldn't it make sense to rescue the trios with few reads?

*Reply: We saw a huge peak in the read-count histogram at 1, the mean read-depth is 24 (pool 4). We filtered out the trios with just one read supporting the trio since the evidence for these trios is the lowest. We show the read-depth histograms before and after one filtering for one of the pools below. We agree that error-correcting these trios would recover some real trios. However, the error-correction is computationally expensive, and throwing out the trios with just one read of support reduces the computational time for error-correction. We have modified the text to indicate the read-count threshold on line 571.*

4. Page 18, line 4: "We found that 99% of 10x cell barcode are more than 6 hamming distances away from each other". Does this mean that 99% of the pairs have a Hamming distance above 6, or that 99% of the barcodes have a Hamming distance above 6 against every other barcode? Or does it mean something else?

*Reply: We have clarified this on line 579.*

5. Page 18, line 17: "all the locBCs are ranked based on abundance". Is this the abundance of the number of reads, the number of UMIs? Another abundance?

*Reply: We have clarified this on line 582 and line 589.*

6. Page 18, line 29: "We filtered out cells that had less than 5 IR intergrations (locBCs) and less than 10 UMIs". Does this mean that a cell with fewer than 5 integrated reporters is systematically thrown away for the rest of the study? This seems implausible since the authors report a mean of 3 integrations per clone at page 8, line 17. Maybe the authors mean that some filtering of the locBCs was performed in the transcriptome? In this case, it is not clear what this means, as counting could be based on the reads, the UMIs, or something else.

*Reply: We are unable to link all the location barcodes to clones using the clonal analysis due to dropout as we discuss in the Methods section “Identifying single cell clones”. Hence the number of integrations per clone detected in this analysis is a subset and smaller than the minimum of 5 reporters per cell that we use as a filter.*

7. Page 18, line 33: "We also filtered out locBCs that were seen in less than 5 cells". The justification that these filters were chosen to maximize reproducibility between replicates would require some data showing that those numbers reach an optimum of reproducibility (to be further defined). Meanwhile, removing locBCs with low expression introduces a bias in the mean. This should be discussed and justified.

*Reply: We tried different parameter combinations and picked the combination of parameters reported in the paper that had higher mean correlation between replicates while retaining most of the locations. Shown below are the mean-correlations for the different parameter combinations that we attempted in Pool 4. Many of the combinations we attempted had similar levels of technical reproducibility indicating the choice of cutoff does not have a huge impact.*

*The mean and variance is calculated for each location so we are potentially removing some lowly expressed locations that are expressed in very few cells, but using this cutoff ensures that the locations we retain and use for downstream modeling have better power to measure mean and variance. We have now discussed this, lines 603-605.*

8. Page 18, line 37: "mean expression was calculated as the average UMI count across all cells that expressed that locBC". The estimator is biased because it does not take into account cells where the locBC is present but not expressed. The authors know which barcodes are present but not expressed in a given cell, as they explain page 8, line 16. The unbiased estimator would be based on a larger sample size, so it seems a better choice to me. The same applies to the estimator of the variance.

*Reply: We agree with the reviewer that knowing which barcodes are present but not expressed would be the unbiased estimator to use. However, due to dropout the clonal analysis recovers only a subset of the total number of clones as we explain in the Methods section “Identifying single cell clones”. We do not have the clonal information for many of the locations and performing the mean/variance analysis on just the locations in the clonal analysis would limit the total number of locations analyzed.*

COMMENTS — nothing to address, just for the sake of scientific discussion.

1. Page 18, line 16-21: Work by Björklund (https://pubmed.ncbi.nlm.nih.gov/27874090/) showed that this variant of sequence clustering seems to be less effective than another one referred to as "message passing". The authors may be interested in some earlier discussion on the design of barcodes and error-correction algorithms.

*Reply: We thank the reviewer for the suggestion.*

2. Page 15, line 57: "For each pool, we performed 2 technical replicates with 8 PCR per replicate". How much input DNA is in each PCR? The weight of one haploid human genome is approximately 35 pg, so for a population of dividing diploid cells, the DNA of one cell is approximately 100 pg. A PCR with 3 ng of input thus corresponds to ~30 cells, carrying at most 150 insertions (based on the assumption of the authors that each cell contains 5 insertions). In practice, inverse-PCR loses at least 2/3 of the insertions because the T4 DNA ligase reaction is far from complete in 16 hours, and some insertions are lost because the restriction fragment is either too large or too small. Thus, a set of 8 PCRs with 3 ng each carries at most ~500 target DNA molecules that can be amplified. For the declared target of 500 insertions per pool, this is a 1x coverage.

It is important to know whether the input in each PCR is above or below 3 ng to know if the coverage is sufficient to discover most of the insertions, or if the mapping explores only a small fraction of the insertions.

*Reply: We thank the reviewer for the comprehensive analysis. For each technical replicate, we start by digesting 4ug of DNA. We do not quantify the DNA after ligation, and simply perform 8 PCR reactions after ligation cleanup. In an ideal world, this should result in 500ng input per PCR. In reality, taking into account the loss of DNA from two column cleanup steps and the ligation inefficiencies, there is probably 100-200ng of DNA in each reaction. In other experiments with inverse PCRs we have also attempted to digest more DNA to increase the complexity of the library and get more insertions, but the reactions tend to be dominated by clones that grow out quicker (and are represented more in the gDNA pool), leading to diminishing returns even if we perform more inverse PCR reactions.*

**Second round of review**

**Reviewer 1**

Thanks to the authors for revising their manuscript and responding to the two Reviewers' queries. Many important missing details were added to the manuscript, substantially improving its readability, and several important analyses were added. However, the response and revisions are largely insufficient to address the concerns brought up by Reviewers, in my opinion. Further, I have several additional points for clarification, detailed below.

Authors' responses to Reviewers:
R1.3: A y-axis label should be added to 3A,B so that readers can understand what they are looking at. I would consider replacing Fig 3A/B with SF3A/B. I find those to be much more compelling. Especially as the units for SF3 are in log2 space, indicating that the distribution of values in linear space has a long tail of high values that likely dominates the mean (R1's concern). Also, SF3 is cut off at the top and the x axis labels of 3F are illegible. For 3F, how are P values derived given that there are N models (LOOCV)?

R1.4: If I understand the response correctly, the one clone containing 27 integrations is accounting for (27*26)/2 = 351 of the points in Fig 4C. The next most abundant clone looks like it accounts for (18*17)/2= 153 of the points. Overall, it appears that most of the points are dominated by few clones. This dependence on few clones and the confounding of extrinsic noise and capture efficiency (see below) make me sceptical of the fractions assigned to extrinsic/intrinsic noise.

R2.Major.1: A citation for this regression-based way of eliminating the relationship between CV and mean would help future readers avoid the same confusion over the validity of this approach. The extremely low reproducibility of the MIN values is somewhat distressing (~4% variance explained from one replicate to the other). "We found that 73% of the locations that were identified as high/low MIN in one replicate were concordant in the other replicate"? does this mean that 73% of those in the top 20% in replicate 1 were also top 20% in replicate 2? Only 4% correspondence is expected by chance, so 73% would be quite good. This does not appear to be the case based on the plot provided in the response (MIN replicate 1 vs 2).

R2.Major.3: LOOCV is much more appropriate than the original analysis. Some details remain opaque. For LOOCV for extrinsic features - are clones being left out, or is it IRs? If IRs, then the same clone is in the train/test data, and would share identical extrinsic features. I believe this could lead to undetected overfitting and inflation of performance. Further, from L662-663 it sounds like the authors tested all motifs for their association with MIN levels across insertion sites, and then used only those that show a significant correlation in subsequent LOOCV models. However, if the correlations were calculated on all insertion sites, this includes the insertion sites in the held out data for each cross-validation fold. Therefore, the feature selection depends subtly on the test data, which can similarly cause undetected overfitting.

R2.Major.4: With a screenshot of (only part of) a results table for motif analysis without headers, it is difficult to determine what, if anything, this means. Are these expected by chance? Even if the original motifs were spurious, you would not expect the same spurious motifs in this shuffled analysis. Much more detail is needed to explain what exactly was tested and what it means here.

R2.Major.6: Unfortunately, I am having to do some guess work on what exactly the authors did here, which should be clarified in the methods (e.g. with equations). It seems like, the mean expression of a barcode L is mean(norm(UMI_L)) where UMI_L is the UMI count for locBC L, and norm(UMI_L) is the fraction of all locBC UMIs for that cell. Though this may be incorrect since the methods say it was normalized to whole transcriptome first, and yet also go on to say that "mean expression was calculated as the average UMI count across all cells that expressed that locBC. Expression variance was calculated as the variance in UMI counts across all cells that expressed that locBC" - not mentioning any such normalization of the counts. The need to correct for capture efficiency suggests that a large component of the "extrinsic noise" is actually technical at the cellular level. This could be a major concern in the analysis of assigning noise to intrinsic/extrinsic fractions.

R2.MI.1: The codebase is quite difficult to navigate and appears to include broken/junk scripts (e.g. "filter_trios.py" is just a bunch of imports with no substance, "stemness_clonal.ipynb" is similar). Having a readme that describes what the scripts do, and how to use them together would be highly beneficial. Once polished in a way that future readers will be able to navigate, the code should be deposited onto Zenodo as well for long term archiving (as GitHub repos can be deleted and modified, and it would be difficult to subsequently reproduce the analysis). Further, the IR construct should be added to AddGene.

R2.Minor.1: R2's comment about "We chose to use the CMV promoter because it should be independent of the cells' regulatory networks and therefore should not be affected by endogenous cellular feedback loops." Appears to have been missed and I agree, this seems tenuous. In particular "independent of the cells' regulatory networks" is highly unlikely since it's an RNA Polymerase II transcribed gene. Further, the authors show that the noise is affected by local histone modifications, indicating that it is not independent. Finally, it is unclear what is meant by "affected by endogenous cellular feedback loops". I would argue that histone modifications/transcription factor binding/transcription form part of a feedback loop. I considered whether it would make sense to mean that the promoter does not affect the regulatory state of the cell, but I don't see how any promoter would significantly impact this, even when present at quite a few copies.

R2.Minor.2: Line 718 and following lines still refer to fluctuation index (instead of CV).

R2.Minor.8, I do not buy the reasoning behind not using the 0-count locBCs. One could infer these are present because they are paired with the same locBCs in other cells, since they are clones. E.g. if you see loc_i and loc_j together in other cells, one could infer that in cells we see only loc_i that loc_j is also there but not expressed.

Other major comments:
SF1: I am unclear how this validation was done and what it means. Is this for clonal cell lines with a single integration? (Otherwise, how could FISH quantify the reporter?) Is all this showing that, when testing the two loci, the same locus was higher in both? (seems like there's a 50:50 chance of this happening by chance.) Further, the FISH data appears to not be mean-normalized, and since the mean is higher the variance should also tend to be higher. I don't know how feasible it is to reproduce the MIN in FISH data, but that would be ideal given that that's what is used in most of the paper. For instance, it's possible that if MIN was calculated for the FISH data, the one with the higher variance actually has a lower MIN. Presumably this would be a lot of FISH experiments and so may not be feasible, and so if my interpretation is right, the authors should consider revising the text to be less conclusive, and if this is truly a coin toss of a validation, consider leaving it out.

L175-6: "We also randomly permuted the mean/MIN labels to determine the significance of the differences we observed". How was this done? How many times? What were you looking for (usually p = fraction of permutations where X was at least as extreme as that observed)?

Fig 6B: Why are there so many very significant p-values that correspond to low fold changes? Are these real? The paper also says there "were no significantly differentially expressed genes once the labels were shuffled", but SF 6B has many P<<0.01 - even more significant than Fig. 6B. It is unclear to me exactly what test is being done here. Regardless, if the P values appear to be so meaningless that they are massively significant when shuffled, why would we believe that they are meaningful when not?

L319: The paper could use some clarification about what the regression results mean. In particular, as the intrinsic features appear to add nothing to performance when extrinsic features are included also. This seems to contradict the earlier findings that there were intrinsic features that could predict MIN. If the model is superior without the intrinsic features, this suggests that the earlier association was spurious.

Minor issues:
In the abstract, the paper claims to have created a "comprehensive genome-wide survey of how genomic locations impact gene expression noise.", which I think is a bit of an overreach for 939 integrations.

The introduction states: "Two other studies have assayed integrations in a high-throughput manner but measured protein levels by flow cytometry rather than mRNA levels22,23. Even for the same reporter gene, noise in translational mechanisms can confound the measurements, especially when trying to understand the impact of features that regulate transcription." The second statement could use a citation and perhaps some clarification since it seems reasonable to assume that identical mRNAs from disparate parts of the genome are translated equally well.

L97-99: "We selected the piggyBac transposon system because it has a bias towards active chromatin regions where transcription is more likely to occur, giving us additional power to study the effects of chromatin on transcriptional noise." It is unclear why this would give additional "power". Does this mean statistical power, and you have more because the ones in repressed loci would be undetectable? (ie. it increases n).

The paper uses "noise" to mean "variance" a lot. I find this confusing as it later comes up with MIN as a better estimate of noise. It might be best to refer to variance as variance.

Double space on line 369.

Fig 6C subtitles are somewhat confusing. Isn't the "control" column from different clones than are listed in the title? Maybe the clone ID should actually be in the "insert" x axis label?

Discussion: "maps of mean expression levels [across the genome] generated by ENCODE." I am unaware of these maps. Is this referring to CAGE data?

Extra parenthesis on Line 313.

Text formatting is inconsistent in at lines 633-635.

**Reviewer 2**

The revisions of the manuscript arrived at a moment when my availability was limited. It has been a challenge for me to find the time to review the new version of the manuscript. I hope that this is not overly detrimental to the Authors.

The Authors addressed my concerns adequately. In many cases, the results were solid and the additional explanations lifted my doubts. In some cases, like for LOOCV, the new results are more robust than in the first version.

In my opinion, two minor issues still require attention. I do not request to check how the Authors address them; I trust that they will do a fine job to further improve the quality of their work.

1. The comment of major point 4. in my first review was not phrased accurately enough. I meant to ask whether the Authors would find motifs with similar strength or statistical significance (as opposed to similar sequences) if they partition the insertion sites at random. I see that the Authors have included some scores next to the motifs in the rebuttal. They could compare those scores to those they obtained when using the high MIN vs low MIN regions. This would allow the reader to make an opinion as to whether the motifs are robust. The mistake was mine: the request was poorly phrased, but I think that this information would improve the quality of the article.

2. The Authors chose not to address minor point 1., which was a request for a citation. It’s acceptable if the citation is hard to find, but in this case, the Authors should use a language that makes it clear that they are speculating, so that readers do not take the comment as a fact, or cite this manuscript as a reference for this information.

While reading the new version, I found a few typos listed below.

Line 191, ofoccurrences (missing space)
Line 313, 75% accuracy using LOOCV). (extra closing bracket).
Line 369, hat there were no (double space)
Line 371, 3 out of 4 IR integrations increases gene expression (increase)

I wish the best of luck to the Authors with their present and future work.

**Authors Response**

**Point-by-point responses to the reviewers’ comments:**

Reviewer #1:
R1.3: A y-axis label should be added to 3A,B so that readers can understand what they are looking at.

*Reply: We have now added a y-axis label.*

I would consider replacing Fig 3A/B with SF3A/B. I find those to be much more compelling. Especially as the units for SF3 are in log2 space, indicating that the distribution of values in linear space has a long tail of high values that likely dominates the mean (R1's concern). Also, SF3 is cut off at the top and the x axis labels of 3F are illegible.

*Reply: We acknowledge the reviewer’s comments, but find Figs 3A/B to be slightly more intuitive. We have fixed the reviewer’s concerns with Supplementary Fig 3.*

For 3F, how are P values derived given that there are N models (LOOCV)?

*Reply: We chose the model with the highest accuracy out of the N models to compute the p-values.*

R1.4: If I understand the response correctly, the one clone containing 27 integrations is accounting for (27*26)/2 = 351 of the points in Fig 4C. The next most abundant clone looks like it accounts for (18*17)/2= 153 of the points. Overall, it appears that most of the points are dominated by few clones.

This dependence on a few clones and the confounding of extrinsic noise and capture efficiency (see below) make me skeptical of the fractions assigned to extrinsic/intrinsic noise.

R2.Major.1: A citation for this regression-based way of eliminating the relationship between CV and mean would help future readers avoid the same confusion over the validity of this approach.

*Reply: We have a citation in the manuscript for this (PMID: 25062021).*

The extremely low reproducibility of the MIN values is somewhat distressing (~4% variance explained from one replicate to the other). "We found that 73% of the locations that were identified as high/low MIN in one replicate were concordant in the other replicate"? does this mean that 73% of those in the top 20% in replicate 1 were also top 20% in replicate 2?

Only 4% correspondence is expected by chance, so 73% would be quite good. This does not appear to be the case based on the plot provided in the response (MIN replicate 1 vs 2).

*Reply: Please refer to our response below Reviewer 2’s further response.*

R2.Major.3: LOOCV is much more appropriate than the original analysis. Some details remain opaque. For LOOCV for extrinsic features - are clones being left out, or is it IRs? If IRs, then the same clone is in the train/test data, and would share identical extrinsic features. I believe this could lead to undetected overfitting and inflation of performance. Further, from L662-663 it sounds like the authors tested all motifs for their association with MIN levels across insertion sites, and then used only those that show a significant correlation in subsequent LOOCV models. However, if the correlations were calculated on all insertion sites, this includes the insertion sites in the held out data for each cross-validation fold. Therefore, the feature selection depends subtly on the test data, which can similarly cause undetected overfitting.

*Reply: Please refer to our response below Reviewer 2’s further response.*

R2.Major.4: With a screenshot of (only part of) a results table for motif analysis without headers, it is difficult to determine what, if anything, this means. Are these expected by chance? Even if the original motifs were spurious, you would not expect the same spurious motifs in this shuffled analysis. Much more detail is needed to explain what exactly was tested and what it means here.

*Reply: Please refer to our response to this comment below (Reviewer 2.1), where Reviewer 2 clarified their question.*

R2.Major.6: Unfortunately, I am having to do some guess work on what exactly the authors did here, which should be clarified in the methods (e.g. with equations). It seems like, the mean expression of a barcode L is mean(norm(UMI_L)) where UMI_L is the UMI count for locBC L, and norm(UMI_L) is the fraction of all locBC UMIs for that cell. Though this may be incorrect since the methods say it was normalized to whole transcriptome first, and yet also go on to say that "mean expression was calculated as the average UMI count across all cells that expressed that locBC. Expression variance was calculated as the variance in UMI counts across all cells that expressed that locBC" - not mentioning any such normalization of the counts. The need to correct for capture efficiency suggests that a large component of the "extrinsic noise" is actually technical at the cellular level. This could be a major concern in the analysis of assigning noise to intrinsic/extrinsic fractions.

*Reply: We have updated the methods with equations and updated the text to clarify this calculation.*

R2.MI.1: The codebase is quite difficult to navigate and appears to include broken/junk scripts (e.g. "filter_trios.py" is just a bunch of imports with no substance, "stemness_clonal.ipynb" is similar). Having a readme that describes what the scripts do, and how to use them together would be highly beneficial. Once polished in a way that future readers will be able to navigate, the code should be deposited onto Zenodo as well for long term archiving (as GitHub repos can be deleted and modified, and it would be difficult to subsequently reproduce the analysis).

*Reply: Please refer to our response below Reviewer 2’s further response.*

Further, the IR construct should be added to AddGene.

*Reply: The IR construct is currently being submitted to AddGene (#83887).*

R2.Minor.1: R2's comment about "We chose to use the CMV promoter because it should be independent of the cells' regulatory networks and therefore should not be affected by endogenous cellular feedback loops." Appears to have been missed and I agree, this seems tenuous. In particular "independent of the cells' regulatory networks" is highly unlikely since it's an RNA Polymerase II transcribed gene. Further, the authors show that the noise is affected by local histone modifications, indicating that it is not independent. Finally, it is unclear what is meant by "affected by endogenous cellular feedback loops". I would argue that histone modifications/transcription factor binding/transcription form part of a feedback loop. I considered whether it would make sense to mean that the promoter does not affect the regulatory state of the cell, but I don't see how any promoter would significantly impact this, even when present at quite a few copies.

*Reply: We have now changed the text to say that we selected CMV because it is a general promoter that will likely respond to different genomic locations (lines 89-91) without asserting independence from endogenous processes.*

R2.Minor.2: Line 718 and following lines still refer to fluctuation index (instead of CV).

*Reply: We have corrected the methods section (lines 715-720).*

R2.Minor.8, I do not buy the reasoning behind not using the 0-count locBCs. One could infer these are present because they are paired with the same locBCs in other cells, since they are clones. E.g. if you see loc_i and loc_j together in other cells, one could infer that in cells we see only loc_i that loc_j is also there but not expressed.

*Reply: We do not have clonal information for the majority of barcodes, because not all the barcodes can be robustly paired or assigned to a clone (as mentioned in our Methods section “Identifying single-cell clones”). In addition, because single-cell data is prone to dropout, we cannot be certain that the lack of counts is due to lack of expression or dropout.*

Other major comments:
SF1: I am unclear how this validation was done and what it means. Is this for clonal cell lines with a single integration? (Otherwise, how could FISH quantify the reporter?) Is all this showing that, when testing the two loci, the same locus was higher in both? (seems like there's a 50:50 chance of this happening by chance.) Further, the FISH data appears to not be mean-normalized, and since the mean is higher the variance should also tend to be higher. I don't know how feasible it is to reproduce the MIN in FISH data, but that would be ideal given that that's what is used in most of the paper. For instance, it's possible that if MIN was calculated for the FISH data, the one with the higher variance actually has a lower MIN. Presumably this would be a lot of FISH experiments and so may not be feasible, and so if my interpretation is right, the authors should consider revising the text to be less conclusive, and if this is truly a coin toss of a validation, consider leaving it out.

*Reply: The FISH was performed on clonal cell lines with a single integration. We show that location 2 has higher mean and variance as measured by both smFISH and SARGENT, though this could, as the reviewer points out, be due to chance. We cannot calculate MIN without being able to do a regression. We have now revised the text to say that the smFISH only qualitatively reproduces the data (lines 124-125).*

L175-6: "We also randomly permuted the mean/MIN labels to determine the significance of the differences we observed". How was this done? How many times? What were you looking for (usually p = fraction of permutations where X was at least as extreme as that observed)?

*Reply: We performed 1000 random permutations of the mean/MIN labels. The p-value was calculated exactly as described by the reviewer.*

Fig 6B: Why are there so many very significant p-values that correspond to low fold changes? Are these real? The paper also says there "were no significantly differentially expressed genes once the labels were shuffled", but SF 6B has many P<<0.01 - even more significant than Fig. 6B. It is unclear to me exactly what test is being done here. Regardless, if the P values appear to be so meaningless that they are massively significant when shuffled, why would we believe that they are meaningful when not?

*Reply: We defined significantly “differentially expressed” genes by thresholding on both fold change and p-value. There are genes with significant p-values, but none of them have large enough fold change.*

L319: The paper could use some clarification about what the regression results mean. In particular, as the intrinsic features appear to add nothing to performance when extrinsic features are included also. This seems to contradict the earlier findings that there were intrinsic features that could predict MIN. If the model is superior without the intrinsic features, this suggests that the earlier association was spurious.

*Reply: We were equally puzzled by the appearing contradiction initially. However, we think this is an expected result given the modeling technique. The intrinsic and extrinsic features are largely independent (epigenetic marks vs global cell states). When we were selecting features for the regression model, we found many intrinsic features with relatively small weights, whereas we had a couple extrinsic features with large weights. When we use intrinsic features alone, we can classify the genomic locations with high and low MIN. But when we combine these features together, we do not have a large enough dataset to allow the model to fully capture the numerous intrinsic features’ contribution to the classification. However, it does not suggest intrinsic features were not predictive but that we may be underpowered to detect the smaller effect sizes of the intrinsic features when the extrinsic features are included.*

Minor issues:
In the abstract, the paper claims to have created a "comprehensive genome-wide survey of how genomic locations impact gene expression noise.", which I think is a bit of an overreach for 939 integrations.

*Reply: We have replaced the word ‘comprehensive’ with extensive’, given that this is the largest number of locations surveyed to our knowledge (line 21).*

The introduction states: "Two other studies have assayed integrations in a high-throughput manner but measured protein levels by flow cytometry rather than mRNA levels22,23. Even for the same reporter gene, noise in translational mechanisms can confound the measurements, especially when trying to understand the impact of features that regulate transcription." The second statement could use a citation and perhaps some clarification since it seems reasonable to assume that identical mRNAs from disparate parts of the genome are translated equally well.

*Reply: We are referring here to stochastic processes in translation that can confound single-cell measurements. We have added a citation.*

L97-99: "We selected the piggyBac transposon system because it has a bias towards active chromatin regions where transcription is more likely to occur, giving us additional power to study the effects of chromatin on transcriptional noise." It is unclear why this would give additional "power". Does this mean statistical power, and you have more because the ones in repressed loci would be undetectable? (ie. it increases n).

*Reply: The word “power” was poorly chosen, we simply meant that we would be able to detect more integrations by scRNA-seq because it is expressed, and that noise in expression is not as relevant for silent regions of the genome. We have clarified this in the text (lines 96-98).*

The paper uses "noise" to mean "variance" a lot. I find this confusing as it later comes up with MIN as a better estimate of noise. It might be best to refer to variance as variance.

*Reply: We have corrected the places where we used ‘noise’ incorrectly.*

Double space on line 369.

*Reply: We have corrected this.*

Fig 6C subtitles are somewhat confusing. Isn't the "control" column from different clones than are listed in the title? Maybe the clone ID should actually be in the "insert" x axis label?

*Reply: We have corrected Fig 6 to make it clearer.*

Discussion: "maps of mean expression levels [across the genome] generated by ENCODE." I am unaware of these maps. Is this referring to CAGE data?

*Reply: We apologize for the error, we were referring to maps generated by other groups (eg. PMID 23953119), but we have removed this line.*

Extra parenthesis on Line 313.

*Reply: We have corrected this.*

Text formatting is inconsistent in at lines 633-635.

*Reply: We have corrected this.*

Reviewer #2:
The revisions of the manuscript arrived at a moment when my availability was limited. It has been a challenge for me to find the time to review the new version of the manuscript. I hope that this is not overly detrimental to the Authors.

The Authors addressed my concerns adequately. In many cases, the results were solid and the additional explanations lifted my doubts. In some cases, like for LOOCV, the new results are more robust than in the first version.

In my opinion, two minor issues still require attention. I do not request to check how the Authors address them; I trust that they will do a fine job to further improve the quality of their work.

1. The comment of major point 4. in my first review was not phrased accurately enough. I meant to ask whether the Authors would find motifs with similar strength or statistical significance (as opposed to similar sequences) if they partition the insertion sites at random. I see that the Authors have included some scores next to the motifs in the rebuttal. They could compare those scores to those they obtained when using the high MIN vs low MIN regions. This would allow the reader to make an opinion as to whether the motifs are robust. The mistake was mine: the request was poorly phrased, but I think that this information would improve the quality of the article.

*Reply: Thank you for the clarification, we now attach screenshots of the discovered motifs by STREME with the associated P- and E-values. We only plot motifs that have p-value < 0.05 (see updated Figure 3). When we compare scrambled motifs (last screenshot pasted below), none of the motifs pass the p-value threshold, suggesting that our motifs are at least weakly associated with high/low MINs respectively.*

Low vs high MIN

High vs low MIN

Scrambled

2. The Authors chose not to address minor point 1., which was a request for a citation. It’s acceptable if the citation is hard to find, but in this case, the Authors should use a language that makes it clear that they are speculating, so that readers do not take the comment as a fact, or cite this manuscript as a reference for this information.

*Reply: We did not find a citation, but have clarified this point in the text and in our response to Reviewer 1.*

While reading the new version, I found a few typos listed below.

Line 191, ofoccurrences (missing space)
Line 313, 75% accuracy using LOOCV). (extra closing bracket).
Line 369, hat there were no (double space)
Line 371, 3 out of 4 IR integrations increases gene expression (increase)

*Reply: We have corrected these typos.*

I wish the best of luck to the Authors with their present and future work.

Reviewer 2's further comments on Reviewer 1's concerns:

R2.Major.1: A citation for this regression-based way of eliminating the relationship between CV and mean would help future readers avoid the same confusion over the validity of this approach.

The extremely low reproducibility of the MIN values is somewhat distressing (~4% variance explained from one replicate to the other). "We found that 73% of the locations that were identified as high/low MIN in one replicate were concordant in the other replicate"? does this mean that 73% of those in the top 20% in replicate 1 were also top 20% in replicate 2? Only 4% correspondence is expected by chance, so 73% would be quite good. This does not appear to be the case based on the plot provided in the response (MIN replicate 1 vs 2).

Referee 2’s comments: The reproducibility is indeed low but one can distinguish an upward trend between the replicates. The Authors probably discretize MIN into top-20% vs bottom-20% because it gives an acceptable reproducibility of 73%. But indeed, looking at the figure provided in the rebuttal, this is hard to believe. It is hard to know where the 20% cut-off is, but if it is 1, say, only 2 points are in the top-right corner, and it the threshold is 0.5, say, it seems that much less than ~3/4 of the points that are above the threshold in one replicate are above the threshold in the other replicate. The authors should provide a plot where the 73% figure appears more clearly, maybe by using different colors or any other device to explain more concretely how this number is obtained.

*Reply: This was an error, the corrected concordance is 33% and the concordant points are highlighted in the plot above. We acknowledge that the reproducibility is not as high as with bulk assays, but was the best we could do given the limitations of scRNA-seq.*

R2.Major.3: LOOCV is much more appropriate than the original analysis. Some details remain opaque. For LOOCV for extrinsic features - are clones being left out, or is it IRs? If IRs, then the same clone is in the train/test data, and would share identical extrinsic features. I believe this could lead to undetected overfitting and inflation of performance. Further, from L662-663 it sounds like the authors tested all motifs for their association with MIN levels across insertion sites, and then used only those that show a significant correlation in subsequent LOOCV models. However, if the correlations were calculated on all insertion sites, this includes the insertion sites in the held out data for each cross-validation fold. Therefore, the feature selection depends subtly on the test data, which can similarly cause undetected overfitting.
Referee 2’s comments: It is indeed more appropriate to leave out the unit that corresponds to a "new experiment". In other words, if a new experiment was performed, it would have to be a new clone, not a new insertion in an existing clone, so Reviewer #1 has a point. For the reader or the user, it is important to know what to expect when performing a "new experiment". The second point is also correct in my opinion. A more robust analysis would try to exclude as many possibilities for "back-door" overfitting as possible.

*Reply: Leaving out specific clones would be difficult to evaluate as each fold that we test would be of a different size. Given the smaller number of IRs that are present in our extrinsic noise analysis it would also be difficult to do the motif analysis separately for each fold, then perform cross-validation. We acknowledge that this could cause some overfitting. As the scale and reproducibility of scRNA-seq continues to improve, the data collected from our method will become more robust.*

R2.Major.4: With a screenshot of (only part of) a results table for motif analysis without headers, it is difficult to determine what, if anything, this means. Are these expected by chance? Even if the original motifs were spurious, you would not expect the same spurious motifs in this shuffled analysis. Much more detail is needed to explain what exactly was tested and what it means here.
Referee 2’s comments: I agree with the point of Reviewer #1. I explained in my review that we probably misunderstood each other with the Authors. The whole point should be clarified.

*Reply: Please refer to Major point 1 above.*

R2.Major.6: Unfortunately, I am having to do some guess work on what exactly the authors did here, which should be clarified in the methods (e.g. with equations). It seems like, the mean expression of a barcode L is mean(norm(UMI_L)) where UMI_L is the UMI count for locBC L, and norm(UMI_L) is the fraction of all locBC UMIs for that cell. Though this may be incorrect since the methods say it was normalized to whole transcriptome first, and yet also go on to say that "mean expression was calculated as the average UMI count across all cells that expressed that locBC. Expression variance was calculated as the variance in UMI counts across all cells that expressed that locBC" - not mentioning any such normalization of the counts. The need to correct for capture efficiency suggests that a large component of the "extrinsic noise" is actually technical at the cellular level. This could be a major concern in the analysis of assigning noise to intrinsic/extrinsic fractions.
 Referee 2’s comments: I tried to see where the methods say "it was normalized to whole transcriptome first" but I could not find it. I could not find which part of the manuscript Reviewer #1 refers to. Lines 607–611 do provide an answer to my original point as to why the logarithm can be negative. The data is substantially transformed (normalized) before analysis, which is typical of datasets with low signal-to-noise ratio.

*Reply: Please see our response to Reviewer 1 above.*

R2.MI.1: The codebase is quite difficult to navigate and appears to include broken/junk scripts (e.g. "filter_trios.py" is just a bunch of imports with no substance, "stemness_clonal.ipynb" is similar). Having a readme that describes what the scripts do, and how to use them together would be highly beneficial. Once polished in a way that future readers will be able to navigate, the code should be deposited onto Zenodo as well for long term archiving (as GitHub repos can be deleted and modified, and it would be difficult to subsequently reproduce the analysis). Further, the IR construct should be added to AddGene.
 Referee 2’s comments: Yes, the code is available but it is not reproducible. This is more up to the Editor to choose where the bar is. When I serve as Editor, I typically ask the Authors to provide a running example and to show how to reproduce the findings of the manuscript (main figures or tables).

*Reply: We have now substantially cleaned up the codebase, added READMEs to folders, and provided scripts to generate each figure, along with the necessary input files. We have also deposited the code to Zenodo -* [*https://zenodo.org/records/10616403*](https://zenodo.org/records/10616403)

R2.Minor.1: R2's comment about "We chose to use the CMV promoter because it should be independent of the cells' regulatory networks and therefore should not be affected by endogenous cellular feedback loops." Appears to have been missed and I agree, this seems tenuous. In particular "independent of the cells' regulatory networks" is highly unlikely since it's an RNA Polymerase II transcribed gene. Further, the authors show that the noise is affected by local histone modifications, indicating that it is not independent. Finally, it is unclear what is meant by "affected by endogenous cellular feedback loops". I would argue that histone modifications/transcription factor binding/transcription form part of a feedback loop. I considered whether it would make sense to mean that the promoter does not affect the regulatory state of the cell, but I don't see how any promoter would significantly impact this, even when present at quite a few copies.
Referee 2’s comments: I agree.

*Reply: Please see our response to Reviewer 1 above.*

R2.Minor.2: Line 718 and following lines still refer to fluctuation index (instead of CV).
Referee 2’s comments: Indeed.

*Reply: Please see our response to Reviewer 1 above.*

R2.Minor.8, I do not buy the reasoning behind not using the 0-count locBCs. One could infer these are present because they are paired with the same locBCs in other cells, since they are clones. E.g. if you see loc_i and loc_j together in other cells, one could infer that in cells we see only loc_i that loc_j is also there but not expressed.
Referee 2’s comments: This point is hard for me to evaluate. I understand what Reviewer #1 means but I am unable to see whether the argument of the Authors is genuine because I do not have access to the data.

*Reply: Please see our response to Reviewer 1 above.*

**Third round of review**

**Reviewer 2**

The Authors have addressed my concerns. Some issues remain, like the lowish reproducibility as discussed in the previous round, but I am still in favor of publishing the manuscript.
